# Protective efficacy of inactivated Newcastle disease virus vaccines prepared in two different oil-based adjuvants

**DOI:** 10.4102/ojvr.v87i1.1865

**Published:** 2020-09-28

**Authors:** Oday A. Aljumaili, Muhammad B. Bello, Swee K. Yeap, Abdul R. Omar, Aini Ideris

**Affiliations:** 1 Department of Veterinary Clinical Studies, Faculty of Veterinary Medicine, Universiti Putra Malaysia, Serdang, Malaysia; 2 Laboratory of Vaccines and Immunotherapeutics, Institute of Bioscience, Universiti Putra Malaysia, Serdang, Malaysia; 3 Department of Veterinary Microbiology, Faculty of Veterinary Medicine, Usmanu Danfodiyo University, Sokoto, Nigeria; 4 Department of Pathology and Microbiology, Faculty of Veterinary Medicine, Universiti Putra Malaysia, Serdang, Malaysia

**Keywords:** Newcastle disease virus, genotype VII, inactivated vaccine, water-in-oil emulsion, virus shedding

## Abstract

Despite the availability of Newcastle disease (ND) vaccines for more than six decades, disease outbreaks continue to occur with huge economic consequences to the global poultry industry. The aim of this study is to develop a safe and effective inactivated vaccine based on a recently isolated Newcastle disease virus (NDV) strain IBS025/13 and evaluate its protective efficacy in chicken following challenge with a highly virulent genotype VII isolate. Firstly, high titre of IBS025/13 was exposed to various concentrations of binary ethylenimine (BEI) to determine the optimal conditions for complete inactivation of the virus. The inactivated virus was then prepared in form of a stable water-in-oil emulsion of black seed oil (BSO) or Freund’s incomplete adjuvant (FIA) and used as vaccines in specific pathogen-free chicken. Efficacy of various vaccine preparations was also evaluated based on the ability of the vaccine to protect against clinical disease, mortality and virus shedding following challenge with highly virulent genotype\VII NDV isolate. The results indicate that exposure of NDV IBS025/13 to 10 mM of BEI for 21 h at 37 °C could completely inactivate the virus without tempering with the structural integrity of the viral hemagglutin-neuraminidase protein. More so, the inactivated vaccines adjuvanted with either BSO- or FIA-induced high hemagglutination inhibition antibody titre that protected the vaccinated birds against clinical disease and in some cases virus shedding, especially when used together with live attenuated vaccines. Thus, genotype VII-based NDV-inactivated vaccines formulated in BSO could substantially improve poultry disease control particularly when combined with live attenuated vaccines.

## Introduction

Newcastle disease (ND) is one of the most important avian diseases that significantly militate against poultry production all over the world (Alexander & Senne 2008). The disease is particularly important in chicken where it inflicts huge economic losses because of high mortality, reduced egg production as well as restrictions in international trade of poultry and poultry products (OIE 2012). The aetiology of the disease is Newcastle disease virus (NDV), a highly pleomorphic enveloped virus that belongs to the genus *Avulavirus* in the family Paramyxoviridae (Nagai, Hamaguchi & Toyoda 1989). The virus encloses a 15.2 kb negative-stranded non-segmented ribonucleic acid (RNA) genome that houses six genes encoding the following structural proteins: nucleocapsid protein (NP), phosphoprotein (P), matrix protein (M), fusion protein (F), hemagglutinin-neuraminidase protein (HN) and large protein (L) (Murulitharan et al. 2013; Yusoff & Tan 2001). Among these proteins, F is considered to be the major molecular determinant of NDV pathogenicity in chickens (De Leeuw et al. 2005). As a rule, virulent NDV strains are known to possess multiple basic amino acid residues (arginine and lysine) between amino acid positions 112–116 and a phenylalanine residue at position 117 in the F protein. On the contrary, isolates of low virulence are considered to be those with monobasic F cleavage site and a leucine residue at position 117 (OIE 2012). In addition, isolates of NDV can be pathotypically classified into lentogenic (mildly virulent), mesogenic (moderately virulent) and velogenic (highly virulent) strains (Bello et al. 2018a; Vijayarani et al. 2010) based on the mean death time (MDT) performed in 9–10-days-old embryonated chicken eggs and the intracerebral pathogenicity index in 1-day-old chicks. Furthermore, NDV is classified into more than 18 phylogenetically distinct genotypes (Bello et al. 2018b; Diel et al. 2012; Snoeck et al. 2013). Interestingly, despite this high genetic diversity, all NDV isolates are serologically grouped into a single serotype because they share a fairly similar immunodominant epitopes (Gogoi, Ganar & Kumar 2017). Thus, any strain of NDV could theoretically elicit immune response capable of cross-protecting against any other strain of the virus.

Over the years, LaSota, B1 and other genotype II-based live attenuated vaccines have been used to curtail the menace of NDV in the global poultry industry (Kapczynski, Afonso & Miller 2013). However, the vaccines have frequently been associated with the severe post-vaccinal respiratory reactions that may predispose the birds to secondary bacterial infections. Furthermore, they can only protect against clinical disease but are unable to block the replication and shedding of phylogenetically divergent virulent isolates especially those belonging to genotype VII taxon (Hu et al. 2011). Currently, members of genotype VII taxon are the most rapidly evolving group of NDV directly linked with the ongoing fourth pandemic of the disease (Miller et al. 2015). They are also widely distributed and have been the most predominantly circulating NDV strains in Malaysia (Roohani et al. 2015) and many other countries over the past two decades (Esmaelizad et al. 2016; Zhang et al. 2012). Effective control of these viruses is therefore of paramount importance in improving the productivity of the global poultry industry.

The current trend in the control of virulent NDV infection is the development of genotype-matched vaccines based on the currently circulating NDV isolates (Bello et al. 2018a). These vaccines have been shown to be more effective not only in disease prevention, but also in blocking the replication and shedding of the virulent virus post-challenge (Bello et al. 2020; Hu et al. 2011; Xiao et al. 2012). While efforts have been put in place to develop live attenuated vaccines based on genotype VII NDV, very few studies exist on genotype-matched inactivated NDV vaccines. Therefore, in the present study, we developed a safe and effective inactivated vaccine based on a recently circulating NDV isolate, formulated it in different adjuvants and evaluated its protective efficacy against the challenge with another virulent genotype VII NDV isolate.

## Materials and methods

### Virus preparations

Newcastle disease virus strain IBS 025/13 is a velogenic isolate with intracerebral pathogenicity index (ICPI) of 1.86. The virus was initially isolated from a well-vaccinated broiler farm in Malaysia and was shown to be a naturally recombinant NDV with NP and P genes derived from genotype II (vaccine strain), while the M, F, HN and L genes being more closely related to genotype VII isolates (Satharasinghe et al. 2016). The virus was propagated in 9-days-old specific pathogen-free (SPF) embryonated chicken eggs and titrated using the methods described by Reed and Muench (1938).

### Virus inactivation

The 0.1 M binary ethylenimine (BEI) was prepared according to the methods of King (1991). Briefly, 0.041 g of 2-bromo-ethylamine HBr (BEA) Sigma (St. Louis, Missouri, United States) was dissolved in 2 mL of 0.175 N NaOH Merck (Darmstadt, Germany) and incubated for 60 min at 37 °C. The BEI solution was then mixed with 10^9.2^ EID_50_ of NDV strain IBS025/13 to make its final concentration of 4 mM and 10 mM in the BEI–virus mixture. The mixture was afterwards incubated in a shaker at 37 °C for 21, 48, 72 and 96 h. Finally, residual BEI was hydrolysed from the samples by sodium thiosulfate (Merck) treatment at the concentration of 10 times of the final BEI concentration.

### Viability test on the inactivated virus

To test the viability of the inactivated virus, two blind passages were performed in 9-days-old SPF embryonated eggs via allantoic cavity inoculation. During each passage, 10^8.6^ EID_50_ of the inactivated virus (NDV IBS025/13) was inoculated into five eggs and embryonic mortality was monitored for 6 days before harvesting allantoic fluid for the next passage or storage (Pharmacopoeia 2008).

### Hemagglutination assay

Hemagglutination assay (HA) titres for live and the inactivated viruses were determined using 1% chicken erythrocytes suspension as described previously (Beard, Hopkins & Hammond 1975). Briefly, 50 *µ*L of allantoic fluids containing either live or inactivated NDV IBS025/13 was added in triplicates to the first wells of 96-well microtitre plate. The allantoic fluid was then diluted in a twofold fashion up to well 11, while the 12th well was left as Phospate buffered saline (PBS) control. Next, 25 *µ*L of 1% chicken red blood cells suspension was added to each well followed by 30 min incubation at room temperature. Hemagglutination assay titre was determined as the highest dilution of the virus that agglutinated 1% chicken erythrocyte suspension was considered the HA titre.

### Gas chromatography mass spectroscopy

*Nigella sativa* seeds (black seed) were purchased from a commercial dealer in Malaysia. Cold pressed method was used in extracting black seed oil (BSO) using manual press machine. The gas chromatography (GC) profile of BSO in this work was obtained using Shimadzu GCMS-QP2010 system. A BP × 5 (5% diphenyl + 95% dimethylpolysiloxane) 30 m (length) × 0.25 mm I.D. with 0.25 *μ*m film thickness was utilised with a flow rate of 0.80 mL/min. A microliter injection was made in split less mode at the temperature of 250 °C. The GC oven was firstly held at 50 °C for a minute and later increased at 3 °C/min to 300 °C; it was again increased at the rate of 10 °C/min to 340 °C. The mass spectrometer was operated in electron power mode at 70 eV. The data were collected from *m/z* 40 to *m/z* 700 (Khalid & Shedeed 2016).

### Preparation of inactivated Newcastle disease virus in oil adjuvant

The inactivated virus was prepared as water-in-oil (W/O) emulsion according to the method of Mady et al. (2013) with slight modification. The oil phase consisted of 28% of oil soluble surfactant Span 80 MP Biomedicals Inc. (Santa Ana, California, United States) mixed with 72% of Freund’s incomplete adjuvant (FIA) Sigma-Aldrich (Schnelldorf, Germany) or BSO, while the water phase was made up of 12% aqueous soluble surfactant Tween 80 (Sigma-Aldrich) mixed with 88% inactivated virus suspension. To prepare the homogenised W/O emulsion, the aqueous phase was added to the oil phase at a ratio of 3:4 in a drop-wise fashion with continuous mixing for 30 min. The hydrophilic lipophilic balance (HLB) was later adjusted to 3 as previously described by Stone et al. (1978).

### Stability test

Stability testing of emulsion involves determination of stability at long-term storage at 4 °C and 25 °C. The test was performed as described by Cessi and Nardelli (1974) and El-Bagoury et al. (2015).

### Vaccination and challenge study

Embryonated SPF eggs were obtained from Veterinary Research Institute (VRI), Ipoh, Malaysia, and hatched in sterile hatchery facility in our laboratory. After hatching, the birds were transferred to the experimental animal facility where they were fed with commercial feeds and allowed to drink water *ad libitum.* A total of 81 birds were randomly divided into seven groups each comprising 11 chickens (with the exception of the first group that had 15 birds that served as negative control). Three groups (groups 2–4) were vaccinated twice subcutaneously at 10 and 24 days with different inactivated NDV vaccine formulations (Table 1). The remaining three groups (groups 5–7) were vaccinated at 10 days old with different inactivated vaccine formulations via subcutaneous route and live NDV vaccine via eye drop (Table 1). At days 7, 14, 21 and 28, serum samples were collected and analysed using hemagglutination inhibition (HI) test as described by Bello et al. (2020). The chickens were later challenged with 10^5^ ELD_50_ of velogenic genotype VII strain IBS002/11 (Roohani et al. 2015) at 38 days old via eye drop. Sera samples were collected from all groups to evaluate the HI titre.

**TABLE 1:** Vaccination of specific pathogen-free chickens with different Newcastle disease virus vaccines.

Group	NDV vaccine	Vaccination regime and dose
1	Control – unvaccinated group	0.5 mL of sterile PBS
2	Inactivated BEI-black seed oil	0.5 mL (10^9.47^ EID50) at day 10 and 24
3	Inactivated BEI-Freund’s incomplete adjuvant	-
4	Inactivated commercial NDV (LaSota) vaccine	0.5 mL (10^8.99^ EID50) at day 10 and 24
5	Inactivated BEI-black seed oil and live commercial NDV vaccine	0.5 mL (10^9.47^ EID50) and live NDV vaccine at day 10
6	Inactivated BEI-Freund’s incomplete adjuvant and live commercial (LaSota) NDV vaccine	-
7	Inactivated and live commercial NDV (LaSota) vaccine	0.5 mL (10^8.99^ EID50) and live NDV vaccine

NDV, Newcastle disease virus; BEI, binary ethylenimine; PBS, phospate buffered saline.

### Pathogenicity scoring system

Chickens were clinically examined and scored on daily basis for 14 days following challenge with the virulent NDV isolate as recommended by Allan and Borland (Allan & Borland 1979). Briefly, chickens were scored 0 if they showed no any clinical symptom, 1 if they had some respiratory symptoms or greenish diarrhoea, 2 when they were moribund and 3 when they were dead. The average scores from each group were measured as group’s total score.

### Virus shedding measurement

Cloacal swab samples were collected from six birds in each group on days 5, 7 and 10 post-challenge (dpc). The samples were transferred into 1 mL sterile PBS, mixed thoroughly and kept at -70 °C until use. Total RNA was extracted from the swab samples using RNeasy^®^ mini kit Plus Qiagen (Hilden, Germany) following manufacturer’s recommendations. Measurement of viral load was performed based on one-step Taqman-based real-time polymersase chain reaction (PCR) as described by Rasoli et al. (2014).

The assay was carried in a final volume of 10 *µ*L consisting of 5 *µ*L iScript™ One-Step RT-PCR Kit for Probes (2× reaction buffer containing 0.25 mM of each deoxynucleotide (dNTP), magnesium ions, iTaq DNA polymerase, stabilisers) (Bio-Rad, United States of America), 1 *µ*L of iScript reverse transcriptase (Bio-Rad), 0.5 *µ*L of 20 mM of forward and reverse primers and 0.5 *µ*L of 5 mM of probe with 3 *µ*L of RNA template, and sterile nuclease-free distilled water was used to top-up to the required volume. The thermocycling programme for the assay was 95 °C for 10 min for initial denaturation followed by 40 cycles of denaturation at 95 °C for 2 min, annealing at 58 °C for 30 s, extension at 72 °C for 15 s and plate read (Rasoli et al. 2014).

### Potency test of Newcastle disease inactivated vaccine

Inactivated ND vaccine formulations that gave the best results were analysed further based on potency tests B as established by the European Pharmacopoeia monograph 870 (Pharmacopoeia 2008). A total of 120 chickens were randomly divided into two groups: BEI-FIA (*n* = 60) and BEI-BSO (*n* = 60). Each group was further subdivided into three sub-groups (20 birds per sub-group) and immunised with 1/25, 1/50 and 1/100 dilution of the vaccines via intramuscular injection. The birds were later challenged with 10^5^ ELD_50_ of NDV strain IBS002/11 via intraocular inoculation. The birds that survived the challenge and showed no any clinical signs were considered protected. The vaccine was considered acceptable if the actual protection was up to 50% based on European Pharmacopoeia monograph (Monograph 0870 2007).

### Ethical considerations

All animal experiments were conducted in accordance with guidelines of the laboratory animal care as approved by the Institutional Animal Care and Use Committee at the Faculty of Veterinary Medicine, Universiti Putra Malaysia (reference no. UPM/IACUC/AUP-R096/2015)

## Results

### Chemical inactivation of Newcastle disease virus IBS025/13

Two different concentrations (4 mM and 10 mM) of BEI were used for virus inactivation. Our results showed that exposure of NDV IBS025/13 to 4 mM of BEI for 21 h could not inactivate the virus, as embryonic mortality was recorded in the inoculated SPF eggs from the first passage. However, when the exposure was extended to 48, 72 and 96 h, no embryonic mortality was recorded at first passage in the inoculated eggs. However, at the second passage, dead embryos were observed 120 h post-inoculation, indicating incomplete inactivation of the virus. On the contrary, incubation of the virus with 10 mM BEI for 21, 48, 72 or 96 h completely inactivated the virus as evidenced by the lack of embryonic mortality at 144-h post-inoculation in both the first and second passages.

### Effect of binary ethylenimine on hemagglutination titre

To test whether exposure to BEI would affect the titre of inactivated virus, the HA test was performed before and after the chemical treatment. Our result showed that BEI treatment only had a mild effect on the HA titre of the inactivated virus which changed from 10 log_2_ to 9 log_2_ (Table 2).

**TABLE 2:** Effect of binary ethylenimine inactivation on the viral hemagglutination assay titre.

Samples	Virus infectivity	HA titre (log_2_)										
		2	4	8	16	32	64	128	256	512	1024	2048
PBS	-	-	-	-	-	-	-	-	-	-	-	-
IBS025	Live	+	+	+	+	+	+	+	+	+	+	-
BEI 21 h	Inactivated	+	+	+	+	+	+	+	+	+	-	-
BEI 48 h	Inactivated	+	+	+	+	+	+	+	+	+	-	-

BEI, binary ethylenimine; HA, hemagglutination assay; PBS, phospate buffered saline.

### Gas chromatography mass spectroscopy analysis of black seed oil

Based on GC–MS analysis, the following constituents were found in BSO: Thujene <alpha-> (3.4%), pinene <alpha-> (0.7%), pinene <beta-> (0.7%), cymene <para-> (7.1%), *trans*-4-methoxy thujane (0.9%), thymoquinone (7.5%), 2,4-decadienal, (E,Z)-(cas) *trans,cis*-2,4-decadienal (1.2%), *trans,trans*-2,4-decadienal (1.9%), n-hexadecanoic acid (6.7%), 5-isopropenyl-2-methyl-7-oxabicyclo[4.1.0]heptan-2-ol (1.8%), 9,12-octadecadienoic acid (z,z)-,methyl ester (cas) methyl linoleate (30%), 9-octadecenoic acid (E)-(26.6%), hexadecanoic acid, 2-hydroxy-1-(hydroxymethyl)ethyl ester (0.5%), E,E,Z-1,3,12-nonadecatriene-5,14-diol (9.3%) and stigmast-5-en-3-ol, (3.beta.)-(cas) 24.beta.-ethyl-5.delta.-,cholesten-3.beta.-ol (0.8%).

### Stability tests

Based on the tests performed in this study, our results showed that the optimised W/O vaccine preparation formed an emulsion that remained intact for more than 3 months at 25 °C or 6 months at 4 °C. This indicates the stability of the inactivated vaccine emulsified in oil phase (data not shown).

### Hemagglutination inhibition test

Post-vaccination antibody titre in the chickens’ sera was determined using HI test with homologous antigens. All birds had no detectable NDV antibody titre just before vaccination. Similarly, the control group showed no HI antibody reactions throughout the study. On the contrary, chickens vaccinated with various ND vaccines showed increasing antibody titre throughout the experimental period. Generally, higher antibody titres were recorded in groups vaccinated with live vaccines compared to those vaccinated with inactivated vaccines. In addition, inactivated vaccines formulated in FIA elicited higher HI titre than those formulated in BSO adjuvant (Table 3).

**TABLE 3:** Geometric mean hemagglutination inhibition titre (log_2_ ± SD) in specific pathogen-free chickens vaccinated with different Newcastle disease vaccines.

Groups	HI antibody titre at different days post-vaccination			
	7	14	21	28
1	1.00 ± 0.00	1.00 ± 0.00	1.00 ± 0.00	1.00 ± 0.00
2	1.00 ± 0.00	3.22* ± 0.50	5.63* ± 1.25	6.19* ± 0.95
3	1.00 ± 0.00	7.00* ± 0.00	7.73* ± 0.50	8.90* ± 1.41
4	1.00 ± 0.00	7.00* ± 0.00	10.00* ± 0.00	10.69* ± 1.25
5	1.18 ± 0.50	7.00* ± 0.00	7.89* ± 1.41	8.73* ± 0.50
6	1.18 ± 0.50	7.00* ± 0.00	8.97* ± 0.81	9.48* ± 0.57
7	1.41* ± 0.57	7.00* ± 0.00	10.24* ± 0.50	10.00* ± 0.00

SPF, specific pathogen-free; ND, Newcastle disease; HI, hemagglutination inhibition test.

* , Values differ significantly are marked with asterisk with p < 0.05 as statistically significant relationship.

### Scoring of mortality, morbidity and pathogenicity

Challenged chickens from all the groups were observed on a daily basis and accordingly scored based on disease severity and mortality. In the control group, all the birds displayed signs of ND, recorded 100% (15/15) mortality and accordingly had the highest average score for ND clinical signs (1.86 ± 0.55) compared to the other groups (data not shown). Chickens in group 2 (those vaccinated with 10^9.47^ EID50 at days 10 and 24) recorded average score of 0.09 ± 0.09. All other groups recorded no mortality or any obvious clinical signs.

### Cloacal virus shedding

Virus shedding from cloaca was determined from all the groups at different time points post-challenge. Our results indicate that virus shedding was observed in all the groups immunised with only the adjuvanted inactivated vaccines (groups 2–4), although differences exist in both the load and duration of the virus shedding among the groups. Interestingly, no virus shedding was observed in groups 5–7 which consisted of chicken vaccinated with various combinations of live and inactivated vaccines. At all the time points, group I had a significantly higher virus shedding than the remaining groups (*p* < 0.05). In fact, at 5 dpc, only group I demonstrated virus shedding (Table 4). At 7 dpc and 10 dpc, groups 2–4 were still showing virus shedding even though the load had begun to decline in comparison to group I (unvaccinated control).

**TABLE 4:** Cloacal virus shedding of specific pathogen-free chickens following challenged with genotype VII Newcastle disease virus.

Groups	Viral copy number at different days post-challenged††		
	Day 5	Day 7	Day 10
1	12.72 ± 0.76 (6/6)†	11.33 ± 0.42 (6/6)	10.72 ± 0.65 (5/6)
2	0.00 ± 0.00* (0/6)	10.4 ± 0.51* (5/6)	9.48 ± 0.21* (3/6)
3	0.00 ± 0.00* (0/6)	9.20 ± 0.16* (3/6)	10.36 ± 0.50 (6/6)
4	0.00 ± 0.00* (0/6)	9.50 ± 0.23* (4/6)	9.28 ± 0.18* (4/6)
5	0.00 ± 0.00* (0/6)	0.00 ± 0.00* (0/6)	0.00 ± 0.00* (0/6)
6	0.00 ± 0.00* (0/6)	0.00 ± 0.00* (0/6)	0.00 ± 0.00* (0/6)
7	0.00 ± 0.00* (0/6)	0.00 ± 0.00* (0/6)	0.00 ± 0.00* (0/6)

† , Swabs were randomly taken from six birds in each group and evaluated to quantify virus shedding. Frequency of birds detected with challenge virus is shown in parenthesis and expressed as the number of positive swabs or total number of swabs tested.

†† , Viral copy number is expressed as Mean ± standard deviation (Log_10_).

### Vaccine potency

Potency outcomes were calculated based on the protection percentage according to Reed and Muench method (Tables 5 and 6) and the results were expressed as protective dose 60 (PD_60_). The vaccination of chickens with diluted BEI–BSO vaccine showed that the protection percentages of chickens inoculated with 0.02 mL (1/25 of the field dose), 0.01 mL (1/50 of the field) and 0.005 mL (1/100 of the field dose) were 95%, 81% and 42%, respectively, and the PD_60_ was 10^−7.612^. In contrast, the vaccination of chickens with diluted BEI–IFA vaccine showed that the protection percentage of chickens inoculated with 0.02 mL (1/25 of the field dose), 0.01 mL (1/50 of the field) and 0.005 mL (1/100 of the field dose) were 98%, 90% and 52%, respectively, and the PD_60_ was 10^−7.532^.

**TABLE 5:** Potency test of binary ethylenimine-inactivated Newcastle disease virus vaccine formulated in black seed oil as adjuvant.

Dilution	Number of protected chickens/Number of chickens	Number of non-protected chickens	Cumulative			% protection
			Protected	Non-protected	Total	
1/25 (10^−8.076^)	18/20	2	45	2	47	95
1/50 (10^−7.775^)	16/20	4	27	4	33	81
1/100 (10^−7.474^)	11/20	9	11	9	26	42

PD_60_ = 7.612923077 = (10^−7.612^)

**TABLE 6:** Potency test of binary ethylenimine–inactivated Newcastle disease virus vaccine formulated in Freund’s incomplete adjuvant.

Dilution	Number of protected chicken/Number of chicken	Number of non-protected chicken	Cumulative			% protection
			Protected	Non-protected	Total	
1/25 (10^−8.076^)	19/20	1	49	1	50	98
1/50 (10^−7.775^)	18/20	2	30	3	33	90
1/100 (10^−7.474^)	12/20	8	12	11	23	52

PD_60_ = 7.532368421 = (10^−7.532^)

## Discussion

Despite the intensive use of LaSota and Hitchner B1 vaccines in poultry, outbreaks of ND are still a common occurrence throughout Southeastern Asia where the most predominantly circulating NDV isolates are taxonomically classified as genotype VII (Roohani et al. 2015). These genotype VII isolates are currently considered to be the most economically important NDV strains in Malaysia because of their huge contribution to the ongoing fourth ND pandemic (Miller et al. 2015). Furthermore, they are also frequently isolated among the farms that have vaccinated using the conventional genotype II-based ND vaccines. As genotype mismatch between the genotype II-based ND vaccines and the circulating genotype VII NDV is considerably believed to be responsible for the sub-optimal protective efficacy of the current vaccines (Miller et al. 2009), development of new vaccines based on the currently prevalent genotype VII NDV stands to improve the effectiveness of ND control in the global poultry industry. Hence, this study was carried out to characterise the efficacy of a chemically inactivated genotype-matched vaccine against genotype VII NDV isolates in Malaysia.

Newcastle disease virus strain IBS025/13 is a highly virulent (MDT 58.5, ICPI 1.68) genotype VII isolate that grows to high titre in chicken-embryonated eggs. In the present study, the virus was successfully inactivated using a chemical compound called BEI. This compound has been shown to inactivate viruses by alkylating their genomic RNA, thereby interfering with the replication process (Delrue et al. 2012). However, when used in excessive quantities, the compound may destroy the structural and immunogenic integrity of the viral surface glycoproteins. Thus, it is always necessary to optimise the conditions for the development of inactivated vaccines using BEI. Based on the results obtained in this study, exposure of NDV strain IBS025/13 to 10 mM of BEI for 21 h at 37 °C completely inactivated the virus. Furthermore, the HA titre of the virus before and after BEI treatment remained fairly similar, signifying that the chemical can effectively inactivate the virus without destroying the viral HN protein. Thus, exposure of virulent NDV to 10 mM of BEI for 21 h at 37 °C could be regarded as the optimal condition for the development of inactivated ND vaccines.

A cardinal requirement for all inactivated vaccines is the need to completely inactivate the entire virus particles in the vaccine preparations. In effect, the inactivation process should render the virus incapable of undergoing any replication and at the same time spare the outer structural proteins of the virus so that neutralising immune response can be elicited in the vaccinated host. This is, however, highly challenging, as some viruses have evolved the strategy of avoiding inactivation by forming virus aggregates, leading to vaccine failure. Thus, all inactivated vaccines are required to be checked for evidence of virus growth after inactivation to avoid the risk of their reversion to virulence. Interestingly, when inactivated IBS025/13 was inoculated into SPF chicken-embryonated eggs in two consecutive passages, no embryonic mortality was recorded even at 6 days post-inoculation. This signifies the complete and stable inactivation of the virus.

Conventionally, inactivated ND vaccines are prepared in commercially available adjuvants such as mineral oil, which are known for their potent immunomodulatory activity. However, in spite of their track record of enhancing the magnitude of immune response to different pathogens, these adjuvants have many drawbacks that limit their prospects in vaccine delivery. For instance, some mineral oils are known to cause severe tissue reactions when used as adjuvants in birds. They may also contain carcinogenic substances that could constitute the risk of developing cancer among the poultry meat consumers. Thus, alternative immunomodulatory agents with enhanced efficacy and minimum toxicity are urgently needed in poultry vaccine industry. Interestingly, recent evidences indicate that *N. sativa* oil, a potent anticancer, anti-inflammatory and antioxidant agent (Sharma et al. 2009) could serve as an excellent adjuvant for a variety of viral pathogens. It is a non-specific immunomodulatory agent that profoundly enhances cytokine gene expression as well as cell-mediated and humoral immune responses (Umar et al. 2015). To investigate its efficacy as an adjuvant for NDV vaccine, we prepared a W/O emulsion of our inactivated NDV in *Nigella sativa* oil and used in it in SPF chicken vaccination.

Our findings reveal a steady rise in HI antibody titres from the point of vaccination up to 4 weeks post-vaccination in all the vaccinated groups, with higher antibody titres generally recorded in groups vaccinated with a combination of live and inactivated vaccines. This is not unexpected as live vaccines are replicating vaccines whose immunogenicity is generally more robust than that the inactivated vaccines (Senne, King & Kapczynski 2004). However, even in the birds vaccinated with inactivated NDV adjuvanted in BSO, we observed a mean HI antibody titre of log_2_ 6.19 ± 0.95, which is known to be above the protective threshold of 5 log_2_ (Kapczynski et al. 2013). Indeed, when combined with live vaccine, inactivated NDV emulsified in BSO induced the mean HI titre of log_2_ 8.73 ± 0.50 which is comparable to the titre in birds vaccinated with commercial NDV vaccine. Thus, given its low toxicity and profound immunomodulatory effects, as established by previous researchers (Salem 2005; Umar et al. 2015; Zaoui et al. 2002), BSO can be efficiently used as an adjuvant for inactivated NDV vaccines especially in combination with the live vaccine.

While all vaccinated birds were protected from overt clinical signs and mortality, virus shedding was noted in all the groups vaccinated with the inactivated vaccines alone. This indicates that these vaccines could only protect against the clinical disease but not against virulent virus infection and replication. Nevertheless, the magnitude and duration of virulent virus shedding in those groups was generally lower than those of the control group whose magnitude of the virus shedding was high from day 5 post-challenge. Similar findings were previously reported (Fentie et al. 2014; Kapczynski & King 2005). Interestingly, virus shedding was not recorded in any of the groups vaccinated with a combination of live and killed vaccines (groups 5–7) throughout the observation period. It is possible that the combination of the two different vaccine preparations robustly stimulates the immune system to produce a strong mucosal immunity that prevents the replication and shedding of the challenged virulent virus.

In conclusion, we have shown that the optimal conditions for chemical inactivation of NDV IBS025/13 are by exposure to BEI at 37 °C for 21 h. These conditions completely inactivate the virus without compromising the structural integrity of the surface glycoproteins of the virus. We have also successfully prepared the inactivated vaccines as stable W/O emulsions (for both BSO and FIA) that elicited strong immune responses against clinical disease and mortality because of virulent genotype VII NDV challenge in chickens. Finally, BSO proved to be an efficient adjuvant as it elicited a comparable immune response with the Freunds incomplete adjuvant and yet reduces vaccine residues and minimises tissue reactions in the animal tissue. This makes it a potential substitute of the widely used mineral oil in adjuvanting inactivated ND vaccines.
